# Warts

**Published:** 1859-03

**Authors:** 


### WARTS.

Warts are removed in a fortnight if creosote is painted on them, and they are then covered with a common sticking plaster, to be renewed every third day. But as creosote is a virulent poison, it is safer to use some acid or strong alkali—say potash or hartshorn, every day, until they disappear, as most of them will, under this latter treatment, if persevered in, using only the creosote in incorrigible cases. We know by personal experience that persevering friction with anything, even with the finger, is efficient in the removal of some kind of warts; and such was Lord Bacon's observation and experience, possibly the vulgar notion, that a wart is cured by stealing a piece of bacon, originated in a hair-brained or muddy headed individual, who used the thing itself instead of the advice of the man who gave it.

